# The efficacy and safety of enzalutamide with trastuzumab in patients with HER2+ and androgen receptor-positive metastatic or locally advanced breast cancer

**DOI:** 10.1007/s10549-021-06109-7

**Published:** 2021-02-16

**Authors:** Andrew Wardley, Javier Cortes, Louise Provencher, Kathy Miller, A. Jo Chien, Hope S. Rugo, Joyce Steinberg, Jennifer Sugg, Iulia C. Tudor, Manon Huizing, Robyn Young, Vandana Abramson, Ron Bose, Lowell Hart, Stephen Chan, David Cameron, Gail S. Wright, Marie-Pascale Graas, Patrick Neven, Andrea Rocca, Stefania Russo, Ian E. Krop

**Affiliations:** 1 NIHR Manchester Clinical Research Facility at The Christie NHS Foundation Trust and Division of Cancer Sciences, School of Medical Sciences, Faculty of Biology, Medicine, and Health, Manchester Academic Health Science Centre, University of Manchester, Manchester, UK; 2 Division of Breast Cancers and Gynecological Tumors, IOB Institute of Oncology, Quironsalud Group, Madrid and Barcelona, Spain; 3 Division of Breast Cancer and Melanoma, Vall d’Hebron Institute of Oncology (VHIO), Barcelona, Spain; 4 Department of Surgery, Centre des Maladies du Sein, CHU de Québec-Université Laval, Québec, Canada; 5 Indiana University Melvin and Bren Simon Cancer Center, Indiana University, Indianapolis, USA; 6 UCSF Comprehensive Cancer Center, University of California, San Francisco, USA; 7 Astellas Pharma Inc., Northbrook, USA; 8 Pfizer Inc., San Francisco, USA; 9 Department of Oncology, Antwerp University Hospital, Edegem, Belgium; 10 Division of Breast Oncology, The Center for Cancer and Blood Disorders, Fort Worth, USA; 11 Vanderbilt-Ingram Cancer Center, Vanderbilt University Medical Center, Nashville, USA; 12 Washington University School of Medicine in St. Louis, Washington University in St. Louis, St. Louis, USA; 13 Division of Medical Oncology, Florida Cancer Specialists & Research Institute, Fort Myers, USA; 14 City Hospital, Nottingham University Hospitals NHS Trust, Nottingham, UK; 15 University of Edinburgh Cancer Research Centre, IGMM, The University of Edinburgh, Edinburgh, UK; 16 Division of Medical Oncology, Florida Cancer Specialists and Research Institute, New Port Richey, USA; 17 Department of Oncology, Le Centre Hospitalier Chrétien (CHC), Liège, Belgium; 18 Department of Gynaecology and Obstetrics, University Hospital Leuven, Leuven, Belgium; 19 Department of Medical Oncology, Istituto Scientifico Romagnolo per lo Studio e la Cura dei Tumori (IRST) IRCCS, Meldola, Italy; 20 Department of Oncology, Udine University Hospital, Udine, Italy; 21 Division of Breast Oncology, Dana-Farber Cancer Institute, 450 Brookline Ave., Boston, MA 02215 USA

**Keywords:** Androgen receptor, Enzalutamide, HER2, Human epidermal growth factor receptor 2, Metastatic breast cancer, Trastuzumab

## Abstract

**Purpose:**

Androgen receptor (AR) expression occurs in up to 86% of human epidermal growth factor receptor 2-positive (HER2+) breast cancers. In vitro*,* AR inhibitors enhance antitumor activity of trastuzumab, an anti-HER2 antibody, in trastuzumab-resistant HER2+ cell lines. This open-label, single-arm, phase II study evaluated the efficacy and safety of enzalutamide, an AR-signaling inhibitor, in patients with advanced HER2+ AR+ breast cancer previously treated with trastuzumab.

**Methods:**

Eligible patients had measurable or non-measurable evaluable disease per Response Evaluation Criteria in Solid Tumors (RECIST) v1.1, Eastern Cooperative Oncology Group status ≤ 1, no history of brain metastases, and previously received ≥ 1 anti-HER2 regimen for advanced disease. Patients received 160 mg oral enzalutamide daily and 6 mg/kg intravenous trastuzumab every 21 days until disease progression or unacceptable toxicity. Primary end point was clinical benefit rate at 24 weeks (CBR24); secondary end points included progression-free survival (PFS) and safety.

**Results:**

Overall, 103 women were enrolled [median age 60 years (range 34–83)]; 62% had received ≥ 3 lines of prior anti-HER2 therapy. CBR24, comprising patients with confirmed partial responses (5%) and durable stable disease at 24 weeks (19%), was 24% in the efficacy evaluable set (*n *= 89). CBR24 did not seem related to AR-expression levels or hormone receptor status. Median PFS was 3.4 months (95% confidence interval 2.0–3.8). Overall, 97 (94%) patients experienced treatment-emergent adverse events (TEAEs), with fatigue most common (34%). Dyspnea (4%) and malignant neoplasm progression (3%) were the only TEAEs grade ≥ 3 reported in ≥ 3 patients. 22 patients (21%) reported serious TEAEs. Four patients (4%) experienced fatal, non-drug-related TEAEs.

**Conclusions:**

Enzalutamide plus trastuzumab was well tolerated, and a subset of patients in this heavily pretreated population had durable disease control. Determination of biomarkers is needed to identify patients most likely to benefit from this combination.

**ClinicalTrials.gov number:**

NCT02091960

**Supplementary Information:**

The online version of this article (10.1007/s10549-021-06109-7) contains supplementary material, which is available to authorized users.

## Introduction

Breast cancer is among the most frequently diagnosed malignancies and the second most common cause of cancer deaths in women worldwide, with an estimated 1.67 million new cases diagnosed globally in 2012 [1]. Breast cancer, including metastatic breast cancer (MBC), is a heterogeneous disease, making its prognosis and management complex. While the 5-year relative survival rate is 99% for patients presenting with localized disease, it is only 27% for patients with MBC [2]. Human epidermal growth factor receptor 2 (HER2) is amplified/overexpressed in approximately 15% of all breast cancer cases [3], making treatment recommendations for MBC highly dependent on hormone receptor (HR) and HER2 status [3, 4]. Survival for patients with HER2-positive (HER2+) MBC has been significantly prolonged due to anti-HER2 therapies [5–9].

The recommended first-line treatment for HER2+ MBC is chemotherapy plus dual HER2 inhibition with trastuzumab and pertuzumab [3, 4]. Trastuzumab emtansine (T-DM1) is recommended over lapatinib plus capecitabine as standard second-line therapy after trastuzumab-based first-line treatment [4]. In practice, the anti-HER2 agent selected depends on country-specific availability, previous anti-HER2 therapy, and time to relapse [4, 10]. However, even with full access to anti-HER2 agents, the vast majority of patients will eventually experience disease progression. Thus, there remains an unmet medical need in HER2+ MBC treatment for effective new therapies [3, 4].

Androgen receptor (AR) expression is observed in up to 86% of HER2+ breast cancers [11–13] and has been investigated as a potential therapeutic target in breast cancer. The AR-signaling inhibitor enzalutamide [14] is either approved or under regulatory consideration for approval for castration-resistant prostate cancer, irrespective of the presence of metastases, and metastatic castration-sensitive prostate cancer (also known as metastatic hormone-sensitive prostate cancer) around the world [15–17]. Enzalutamide enhances the in vitro antitumor activity of trastuzumab in trastuzumab-resistant HER2+ cell lines [18], warranting clinical investigation of whether inhibiting AR in HER2+ breast cancers in combination with currently available anti-HER2 therapeutics could improve patient outcomes. We therefore evaluated the efficacy and safety of combining the anti-HER2 therapy trastuzumab with enzalutamide in patients with HER2+ AR+ locally advanced breast cancer or MBC.

## Patients and methods

### Study design

This was a multinational, multicenter, open-label, single-arm, two-stage, phase II trial evaluating the efficacy, safety, and tolerability of enzalutamide in combination with trastuzumab (NCT02091960). The study was approved by an independent ethics review board at each participating site or by national authorities and was conducted according to the provisions of the Declaration of Helsinki and the Good Clinical Practice Guidelines of the International Council for Harmonisation of Technical Requirements for Registration of Pharmaceuticals for Human Use.

### Study population

Eligible patients were women aged ≥ 18 years with histologically or cytologically proven HER2+ AR+ breast carcinoma (see Online Resource 1: Patients and methods). Inclusion in the study could be based on local or central pathology assessment. Patients with locally assessed breast cancer also had their samples sent for central pathology laboratory assessment. Patients were allowed to remain in the study if subsequent central assessment did not confirm locally assessed AR+ breast carcinoma.

Inclusion criteria of the study included the following: (i) metastatic or locally advanced disease that was not amenable to curative treatment and had to have measurable or non-measurable, evaluable disease per Response Evaluation Criteria in Solid Tumors (RECIST) v1.1 [19], (ii) previous treatment with ≥ 1 line of anti-HER2 therapy in the metastatic or locally advanced disease setting, (iii) documented progression or discontinued the most recent anti-HER2 therapy due to investigator decision or toxicity other than cardiotoxicity, and (iv) an Eastern Cooperative Oncology Group performance status ≤ 1 and a minimum life expectancy of ≥ 6 months. Exclusion criteria included (i) severe concurrent disease, (ii) severe infection or significant comorbidity, (iii) known or suspected brain metastases or active leptomeningeal disease, (iv) a history of a non-breast-cancer malignancy, (v) inadequate marrow, hepatic, and/or renal function, (vi) a history of seizures, and (vii) clinically significant cardiovascular disease.

### Analysis sets

The safety analysis set (SAF) included all enrolled patients who received at least one or a partial dose of study treatment. The full analysis set (FAS) was defined as all patients in the SAF who had centrally assessed AR+ breast cancer (defined as ≥ 10% of tumor cells with nuclear expression). The efficacy evaluable set (EES) included all patients in the FAS who had at least one available post-baseline tumor assessment. The primary analysis was performed in the EES, while all efficacy analyses were performed in both the EES and FAS. Patient disposition and safety were based on SAF.

### Treatments

Patients received a once-daily oral dose of 160 mg enzalutamide (4 × 40 mg capsules) and trastuzumab, starting with a loading dose (8 mg/kg) followed by either intravenous (6 mg/kg) or subcutaneous (600 mg/5 mL) administration every 21 days. Dose interruptions or modifications of enzalutamide and trastuzumab were permitted due to toxicity, as defined in the study protocol (see Online Resource 1: Patients and methods). Patients continued on treatment until disease progression, unacceptable toxicity, or any other discontinuation criteria were met.

### Study end points

Primary end point was clinical benefit rate at 24 weeks (CBR24), defined as the proportion of evaluable patients with best objective response of confirmed complete response (CR) or partial response (PR) per RECIST v1.1 or prolonged stable disease (SD) ≥ 24 weeks. Key secondary end points were best overall response rate (BORR; CR or PR), overall response rate (ORR; CR or PR) at 24 weeks, progression-free survival (PFS), time to progression (TTP), time to response (TTR), and safety. Prespecified exploratory end points included CBR24 in subgroups by AR-expression levels, hormone receptor (HR) status, and lines of prior therapy.

### Assessments

Radiographic disease assessments according to RECIST v1.1 [19] were performed by the investigator at baseline and every 8 weeks up to week 49, then every 12 weeks thereafter. Local AR testing results were confirmed centrally using the Ventana Assay (Ventana Medical Systems, Inc., Tucson, USA) (see Online Resource 1: Patients and methods). Safety assessments throughout the study included the recording of adverse events (AEs). Cardiac safety assessments were required throughout the study to monitor for trastuzumab-associated cardiotoxicity (see Online Resource 1: Patients and methods).

### Statistical analysis

The study followed an optimal Simon’s two-stage design to determine sample sizes. A CBR24 of ≥ 3 out of 21 evaluable patients was required in stage I to continue to stage II. In total, approximately 80 patients were planned to be enrolled to achieve a dataset with at least 66 evaluable AR+ patients. The null hypothesis that the true CBR24 is ≤ 10% was tested against a one-sided alternative at a 5% significance level. This design has a statistical power of 90% when the true CBR24 is 25%. Patients in the FAS were included in primary and secondary efficacy analyses. See Online Resource 2: Statistical analysis for descriptive statistics. CBR, BORR, and ORR are summarized, including 95% two-sided exact confidence intervals (CIs) (Clopper–Pearson method). Kaplan–Meier analyses were used to estimate the median PFS, TTP, and TTR.

## Results

This trial was conducted in 35 centers in six countries (Belgium, Canada, Italy, Spain, United Kingdom, and United States). Between September 2014 and August 2016, 103 patients were enrolled and received at least one dose of enzalutamide and trastuzumab (see Online Resource 3: Fig. S1). The actual enrollment was greater than the planned enrollment goal of 80 patients due to increased screening and patient recruitment. At the end of stage I, six out of 22 evaluable patients (27%; 95% CI 10.7–50.2) demonstrated CBR24, thus meeting the prespecified requirement for the study to continue to stage II.

Baseline patient demographics and disease characteristics are reported in Table 1. In the efficacy evaluable set, approximately 52% of patients were HR + and 87% were perimenopausal or post menopausal. All patients had been previously treated with trastuzumab, and 62% had received ≥ 3 lines of prior anti-HER2 therapy. HER2 status was locally determined. Local AR testing results were confirmed centrally using the Ventana Assay, with a concordance level of 98.6% (see Online Resource 4: Table S1). AR staining was high (i.e., 50–100% positive cells) in approximately 90% of patients.

**Table 1:** Patient demographics and baseline characteristics

	Safety analysis set^a^ (*n *= 103)	Full analysis set^b^ (*n* = 96)	Efficacy evaluable set^c^ (*n* = 89)
Age (years)			
Median (range)	60.0 (34–83)	60.0 (34–83)	60.0 (34–83)
Age categories (years), *n* (%)			
≤ 65	78 (76)	73 (76)	66 (74)
66–75	18 (18)	17 (18)	17 (19)
> 75	7 (7)	6 (6)	6 (7)
BMI (kg/m^2^)			
Median (range)	25.7 (14–50)	25.7 (14–50)	25.6 (14–50)
Ethnicity, *n* (%)			
Not Hispanic or Latino	98 (95)	91 (95)	84 (94)
Hispanic or Latino	5 (5)	5 (5)	5 (6)
Race, *n *(%)			
White	90 (87)	83 (87)	78 (88)
Black or African-American	8 (8)	8 (8)	6 (7)
Asian	3 (3)	3 (3)	3 (3)
Other	2 (2)	2 (2)	2 (2)
ECOG performance status at baseline, *n* (%)			
0	51 (49)	49 (51)	47 (53)
1	51 (49)	46 (48)	42 (47)
Unknown	1 (1)	1 (1)	0
Time from initial diagnosis to enrollment (days)			
* n*	95	89	83
Median; minimum, maximum	1199; 30, 4713	1321; 30, 4713	1340; 30, 4713
HER2 status, *n* (%)^d^			
Positive	89 (86)	83 (86)	77 (87)
Negative	2 (2)	2 (2)	2 (2)
Unknown	12 (12)	11 (12)	10 (11)
HER2 testing method confirming HER2 status, *n* (%)^d^			
Immunohistochemistry	35 (34)	33 (34)	30 (34)
In situ hybridization	27 (26)	25 (26)	24 (27)
HER2 amplification	22 (21)	21 (22)	19 (21)
Unknown	19 (18)	17 (18)	16 (18)
HR status, *n* (%)			
Positive^e^	51 (49)	48 (50)	46 (52)
Negative	38 (37)	35 (36)	31 (35)
Unknown	14 (14)	13 (14)	12 (14)
AR + from Ventana Assay, *n* (%)			
> 0– < 10%	2 (2)	0	0
10– < 50%	8 (8)	8 (8)	7 (8)
50–100%	88 (90)	88 (92)	82 (92)
Unknown	5	0	0
Lines of prior antineoplastic therapy, *n* (%)^f^			
1	14 (13)	13 (14)	13 (15)
2	20 (19)	17 (18)	15 (17)
3	11 (11)	10 (10)	10 (11)
4	12 (12)	12 (12)	11 (12)
> 4	46 (45)	44 (46)	40 (45)
Lines of prior anti-HER2 therapy, *n* (%)			
1–2	NA	NA	33 (37)
3–4	NA	NA	24 (27)
≥ 5	NA	NA	31 (35)
Menopausal status,^g^ *n* (%)			
Premenopausal	13 (12)	12 (12)	12 (13)
Perimenopausal	13 (12)	12 (12)	12 (13)
Post menopausal	77 (76)	72 (76)	65 (74)

*AR* androgen receptor, *BMI* body mass index, *ECOG* Eastern Cooperative Oncology Group, *ER* estrogen receptor, *FISH* fluorescence in situ hybridization, *HER2* human epidermal growth factor receptor 2, *HR* hormone receptor, *NA* data not available, *PgR* progesterone receptor

^a^All enrolled patients who received at least one or a partial dose of study treatment

^b^All patients in the safety analysis set who had centrally assessed AR+ breast cancer (defined as ≥ 10% of tumor cells with nuclear expression)

^c^All patients in the full analysis set who had at least one available post-baseline tumor assessment

^d^Local HER2 testing method from most recent biopsy (all patients had at least one biopsy with HER2+ status)

^e^Positive HR status = ER+ and PgR+ or ER− and PgR+ or ER+ and PgR−

^f^Includes all therapies in the settings of locally advanced and metastatic disease and recurrence. It excludes adjuvant and neoadjuvant therapy

^g^Post-menopausal status was defined as no spontaneous menses for ≥ 12 months with FISH > 40 IU/L for patients aged < 55 years, documented surgically sterile, or ≥ 1 month post hysterectomy prior to screening

The median duration of exposure for enzalutamide was 70 days (range 1–660). Patients received a median of four trastuzumab infusions (see Online Resource 5: Table S2). At the time of data cut-off (February 2017), 12 (12%) patients remained on treatment.

## Efficacy

**Fig. 1:**
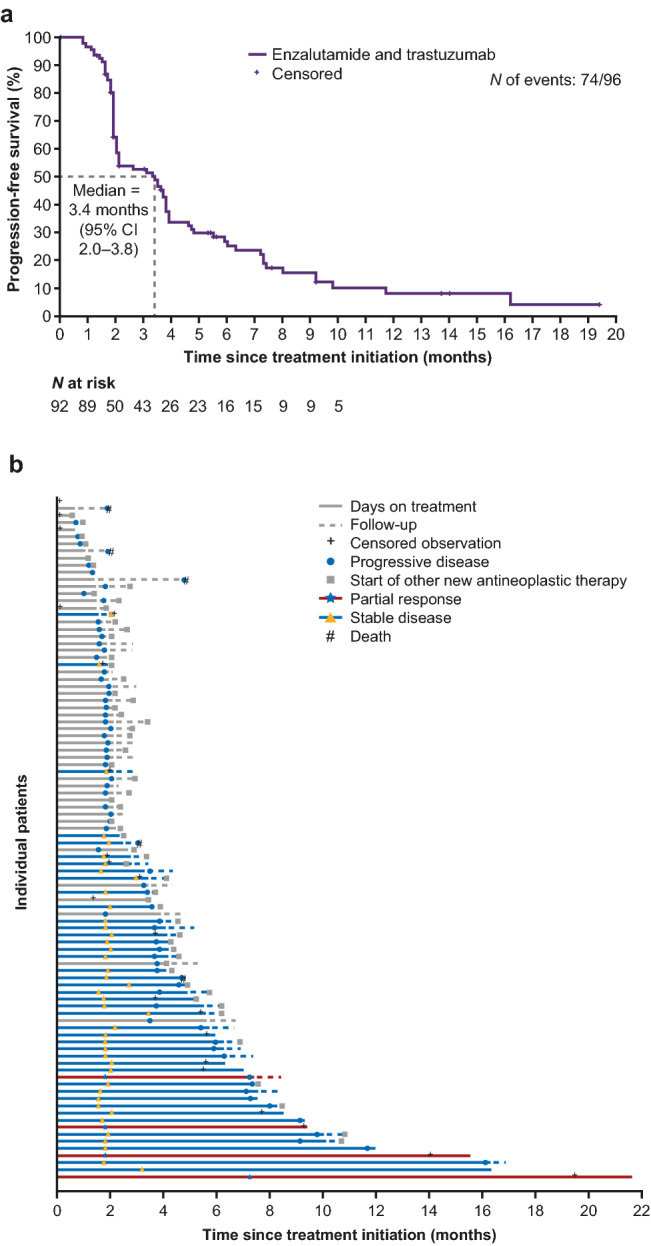
**a** Kaplan–Meier curve of progression-free survival^a^ in the full analysis set; **b** swimmer plot of response to treatment for individual patients ^a^Progression-free survival is defined as the time from the date of first dose of enzalutamide until the date of disease progression per RECIST v1.1 or death from any cause on study (death within 168 days after treatment discontinuation), whichever occurred first *CI* confidence interval, *RECIST* Response Evaluation Criteria in Solid Tumors

In the primary efficacy analysis set, CBR24 was 24% (21/89 patients) (Table 2). Four (5%) patients had confirmed PR and 17 (19%) patients had durable SD at 24 weeks. Additionally, 42 (47%) patients had a best overall response of SD. BORR was 5% and ORR at 24 weeks was 3%. Median TTR and TTP were 57.0 days (range 57–222) and 108.0 days (95% CI 61–116), respectively. Median PFS was 3.4 months (95% CI 2.0–3.8) (Fig. 1a). A plot of response to treatment for individual patients is shown in Fig. 1b.

**Table 2:** Tumor response

Efficacy end point	Efficacy evaluable set^a^ (*n* = 89)	Full analysis set (*n* = 96)
CBR24 (CR or PR or prolonged SD > 24 weeks), *n* [% (95% CI)]	21 [23.6 (15.2–33.8)]	21 [21.9 (14.1–31.5)]
Best overall response,^b^ *n* (%)		
CR	0 (0)	0 (0)
PR	4 (5)	4 (4)
SD	42 (47)	42 (44)
Durable SD at ≥ 24 weeks^c^	17 (19)	17 (18)
Progressive disease	42 (47)	42 (44)^d^
Not evaluable	1 (1)	1 (1)^e^
Best overall response rate (CR or PR), *n* [% (95% CI)]	4 [4.5 (1.2–11.1)]	4 [4.2 (1.1–10.3)]
Overall response rate at 24 weeks (CR or PR), *n* [% (95% CI)]	3 [3.4 (0.7–9.5)]	3 [3.1 (0.6–8.9)]
Time to progression (days),^f^ median (95% CI)	108.0 (61–116)	108.0 (61–116)
Time to response (days),^g^ median (range)	57.0 (57–222)	57.0 (57–222)

*CBR24* clinical benefit rate at 24 weeks, *CI* confidence interval, *CR* complete response, *FAS* full analysis set, *PR* partial response, *RECIST* Response Evaluation Criteria in Solid Tumors, *SD* stable disease

^a^Primary efficacy analysis set

^b^Best overall response per RECIST v1.1 (CR, PR, SD, progressive disease, and not evaluable)

^c^Durable SD is a subset of SD

^d^Excludes 5 of 7 patients in the FAS with progressive disease but no post-baseline tumor radiographic assessments

^e^Excludes 7 patients without post-baseline tumor assessments who are in the FAS

^f^Time to progression is defined as the time from the first date of enzalutamide treatment until the date of disease progression per RECIST v1.1

^g^Time to response is defined as the time from the first date of enzalutamide treatment to initial CR or PR

### Exploratory efficacy subgroup analyses

In exploratory analyses of efficacy in subgroups defined by AR-expression level (percentage of tumor cells with nuclear expression) and by HR status (HR + or HR−), CBR24 was similar in all subgroups versus the overall patient population (see Online Resource 6: Table S3). Additionally, efficacy did not appear to be affected by the number of previous lines of antineoplastic therapy or anti-HER2 therapy (see Online Resource 7: Table S4).

## Safety

Treatment-emergent adverse events (TEAEs) of any grade were reported in 97 (94%) patients; the most common (≥ 10%) included fatigue in 35 (34%) and nausea in 28 (27%) patients (Table 3). Dyspnea (four patients = 4%) and malignant neoplasm progression due to breast cancer (three patients = 3%) were the only TEAEs of grade ≥ 3 reported in ≥ 3 patients. Serious TEAEs were reported in 22 (21%) patients; the most frequent (≥ 2.0%) were malignant neoplasm progression due to breast cancer (five patients = 5%), nausea (three patients = 3%), and vomiting (three patients = 3%). Four patients (4%) experienced fatal TEAEs: (i) two malignant neoplasm progression, (ii) one pulmonary edema, and (iii) one general physical health deterioration associated with concurrent worsening abdominal pain and dyspnea. None of these fatal AEs were assessed as related to a study drug.

**Table 3:** Treatment-emergent adverse events

Event, *n* (%)	Safety analysis set (*n* = 103)
Total number of TEAEs	97 (94)
Serious TEAEs	22 (21)
Enzalutamide- or trastuzumab-related TEAEs	78 (76)
Enzalutamide-related TEAEs	75 (73)
Trastuzumab-related TEAEs	39 (38)
Serious (enzalutamide-related) TEAEs	3 (3)
TEAE leading to permanent discontinuation of enzalutamide or trastuzumab	21 (20)
Study-drug-related TEAEs leading to permanent discontinuation of enzalutamide	5 (5)
Study-drug-related TEAEs leading to permanent discontinuation of trastuzumab	4 (4)
Deaths	4 (4)
Any grade TEAE (≥ 10% patients)^a,b^	
Fatigue	35 (34)
Nausea	28 (27)
Hot flush	17 (17)
Decreased appetite	15 (15)
Dyspnea	15 (15)
Back pain	14 (14)
Dizziness	14 (14)
Headache	14 (14)
Constipation	13 (13)
Diarrhea	13 (13)
Arthralgia	12 (12)
Pain in extremity	11 (11)
Vomiting	11 (11)
Grade ≥ 3 (≥ 2 patients)^a,b^	
Dyspnea	4 (4)
Abdominal pain	2 (2)
Back pain	2 (2)
Fatigue	2 (2)
Malignant neoplasm progression–breast cancer	3 (3)
Pneumonia	2 (2)
Thrombocytopenia	2 (2)
Vomiting	2 (2)

*NCI CTCAE* National Cancer Institute Common Terminology Criteria for Adverse Events, *TEAE* treatment-emergent adverse event

^a^NCI CTCAE grade (v4.03)

^b^By preferred term

Enzalutamide- or trastuzumab-related TEAEs occurred in 78 (76%) patients (Table 3). Eight serious enzalutamide-related TEAEs, nausea (two events), vomiting, diarrhea, dyspepsia, asthenia, accidental overdose, and dyspnea, were reported in three (3%) patients; no trastuzumab-related serious TEAEs were reported. Drug-related TEAEs necessitating dose reduction of enzalutamide occurred in seven (7%) patients (see Online Resource 5: Table S2), the most frequent being fatigue (three patients = 3%). Study-drug-related TEAEs led to permanent discontinuation of enzalutamide in five (5%) and of trastuzumab in four (4%) patients (Table 3).

## Discussion

In this single-arm phase II study of enzalutamide plus trastuzumab in heavily pretreated patients with advanced HER2+ AR+ breast cancer, CBR24 was 24% in the primary analysis set, with a BORR of 5%. Overall, a median PFS of 3.4 months was observed.

Direct comparisons cannot be made across different clinical studies due to the heavily pretreated nature and specific AR+ subset of the HER2+ MBC patient population in this study, for which equivalent data are scarce. In a study with HER2+ locally advanced/MBC patients who had received a median of three trastuzumab regimens, lapatinib in combination with trastuzumab showed an ORR of 10.3% compared to 6.9% for lapatinib alone [6]. In the TH3RESA study of T-DM1 in patients who had previously received ≥ 2 HER2-directed regimens in the advanced setting, including trastuzumab and lapatinib, median PFS (T-DM1 = 6.2 months versus physicians’ choice = 3.3 months) was higher than in the current study for T-DM1, although the patient populations are not directly comparable [20]. Overall, the combination of trastuzumab plus enzalutamide appears to offer durable disease control in a subset of patients with heavily pretreated HER2+ AR+ MBC; however, the clinical impact of this observation is limited because those patients most likely to benefit could not be identified in advance.

The interpretation of these efficacy results should take into account the heavily pretreated patient population in this study (> 60% having received ≥ 3 previous lines of anti-HER2 therapy), although the number of previous lines of anti-HER2 therapy did not appear to be associated with CBR24. In this study, AR-expression levels and HR status did not appear to predict benefit of the combination. However, preclinical data suggest that AR plays a differential role in tumor suppression and oncogenesis within ER+ and ER− breast tissue, respectively [21]. The use of more sophisticated analyses of endocrine signaling may reveal the interaction of HR status and AR in HER2+ MBC in future trials. Current treatments for HER2+ MBC are based on anti-HER2 therapies [3, 4]; however, there remains a need for new targeted treatments with predictive biomarkers to identify patient subgroups that are most likely to respond, including patients with HER2+ AR+ MBC. Indeed, multiple new treatments are being evaluated for HER2+ MBC, including cyclin D-dependent kinase 4/6 inhibitors, tyrosine kinase inhibitors, phosphoinositide 3-kinase inhibitors, mammalian target of rapamycin inhibitors, immunotherapies, antibody drug conjugates, monoclonal antibodies, and therapeutic dendritic cell-based vaccines [22, 23].

In this study, enzalutamide showed a favorable safety profile, consistent with that seen in men with prostate cancer [17] and women with AR-expressing, triple-negative breast cancer [24]. No new safety signals were observed in this female breast cancer population. The frequencies of the most common enzalutamide-related TEAEs in this study, fatigue (30%) and nausea (20%), were in line with those reported in previous enzalutamide trials in men.

This study had limitations. It was a single-arm study consisting of a heterogenous population and, consequently, direct comparison of the efficacy and safety results of enzalutamide plus trastuzumab with other therapies is not possible. Moreover, information that may have assisted in the exploratory analyses to identify predictive biomarkers, such as further details of local genetic testing (e.g., HER2 gene copy number and fluorescence in situ hybridization ratio) and definitions of local estrogen receptor and/or progesterone receptor positivity, were not collected. Importantly, we were unable to centrally assess 24 locally reviewed AR+ cases and 11 HER2+ cases due to a lack of sample material.

## Conclusions

The combination of enzalutamide and trastuzumab was well tolerated, and a subset of patients derived durable disease control. Determination of biomarkers to identify patients most likely to benefit from this combination are needed for this intervention to have a meaningful clinical impact.

## Supplementary Information

Below is the link to the electronic supplementary material.

Supplementary material 1 (PDF 126 kb)

## Data Availability

Access to anonymized individual participant level data collected during the study, in addition to supporting clinical documentation, is planned for studies conducted with approved product indications and formulations, as well as compounds terminated during development. Studies conducted with product indications or formulations that remain active in development are assessed after study completion to determine if Individual Participant Data can be shared. Conditions and exceptions are described under the Sponsor Specific Details for Astellas on www.clinicalstudydatarequest.com. Study-related supporting documentation is redacted and provided if available, such as the protocol and amendments, statistical analysis plan, and clinical study report. Access to participant level data is offered to researchers after publication of the primary manuscript (if applicable) and is available as long as Astellas has legal authority to provide the data. Researchers must submit a proposal to conduct a scientifically relevant analysis of the study data. The research proposal is reviewed by an Independent Research Panel. If the proposal is approved, access to the study data is provided in a secure data sharing environment after receipt of a signed Data Sharing Agreement.

## Abbreviations

AE: Adverse event
AR: Androgen receptor
AR+: Androgen receptor-positive
BMI: Body mass index
BORR: Best overall response rate
CBR24: Clinical benefit rate at 24 weeks
CI: Confidence interval
CR: Complete response
ECOG: Eastern Cooperative Oncology Group
EES: Efficacy evaluable set
ER: Estrogen receptor
FAS: Full analysis set
FISH: Fluorescence in situ hybridization
HER2: Human epidermal growth factor receptor 2
HER2+: Human epidermal growth factor receptor 2-positive
HR: Hormone receptor
MBC: Metastatic breast cancer
NA: Data not available
NCI CTCAE: National Cancer Institute Common Terminology Criteria for Adverse Events
ORR: Overall response rate
PFS: Progression-free survival
PgR: Progesterone receptor
PR: Partial response
RECIST: Response Evaluation Criteria in Solid Tumors
SAF: Safety analysis set
SD: Stable disease
T-DM1: Trastuzumab emtansine
TEAE: Treatment-emergent adverse event
TTP: Time to progression
TTR: Time to response
