# Supplementary material for: The efficacy and safety of enzalutamide with trastuzumab in patients with HER2+ and androgen receptor-positive metastatic or locally advanced breast cancer
Source: Breast Cancer Res Treat. 2021 Feb 16;187(1):155–65. doi: 10.1007/s10549-021-06109-7 (PMC8062601; doi:10.1007/s10549-021-06109-7)
Supplement: Supplementary file 1 — Supplementary material 1 (PDF 126 kb) [file 10549_2021_6109_MOESM1_ESM.pdf]

Supplementary material

Breast Cancer Research and Treatment

The efficacy and safety of enzalutamide with trastuzumab in patients with HER2+ and androgen receptor-positive metastatic or locally advanced breast cancer

A. Wardley • J. Cortes • L. Provencher • K. Miller • A. J. Chien • H. S. Rugo • J. Steinberg • J. Sugg • I. C. Tudor • M. Huizing • R. Young • V. Abramson • R. Bose • L. Hart • S. Chan • D. Cameron • G. S. Wright • M.-P. Graas • P. Neven • A. Rocca • S. Russo • I. E. Krop*

**Correspondence to*: Dr. Ian E. Krop, Division of Breast Oncology, Dana-Farber Cancer Institute, Boston, USA. E-mail: ian_krop@dfci.harvard.edu

Online Resource 1

Patients and methods

Human epidermal growth factor receptor 2-positive (HER2+) definition

HER2+ was defined as a score of 3+ for staining by immunohistochemistry (IHC), or IHC 2+ with HER2 gene amplification as determined by a locally approved *in situ* hybridization (ISH) assay, or (for patients without IHC data) HER2 gene amplification.

Androgen receptor (AR) testing and enrollment

Patients had AR+ breast cancer, defined as any tumor cells with nuclear AR staining by IHC. Enrollment was based on the local pathologist's findings of AR positivity. Additionally, tissue was sent to a central pathology laboratory for confirmatory AR+ assessment using the Ventana AR Assay (Ventana Medical Systems, Inc., Tucson, USA), using anti-AR (SP107) rabbit monoclonal primary antibody. If central assessment could not confirm AR+ disease, the patient was permitted to remain in the study.

Treatments and dose modifications

A loading dose of 8 mg/kg trastuzumab was administered on day 1 for patients whose last dose of trastuzumab was >21 days prior (or who were on weekly trastuzumab for <2 weeks).

Interruption of enzalutamide treatment was permitted for patients who experienced a grade ≥3 toxicity that was attributed to enzalutamide and could not be ameliorated with adequate medical intervention. Enzalutamide may have been resumed at the original dose or at a reduced dose (80 mg/daily or 120 mg/daily) per investigator discretion. Treatment interruption for up to 4 weeks must have been discussed and approved with the medical monitor. Enzalutamide interruption for >4 weeks had to be approved by the medical monitor;

otherwise, the patient was discontinued. A dose adjustment was also considered in patients with grade 2 toxicities that interfered with quality of life.

Dose modifications for infusion reaction to trastuzumab were managed according to institution standard. If a patient experienced cardiomyopathy, trastuzumab was withheld for at least 4 weeks if either of the following occurred: (i) a ≥16% absolute decrease in left ventricular ejection fraction (LVEF) from baseline value or (ii) LVEF below institutional limit of normal and ≥10% absolute decrease in LVEF from baseline value. Trastuzumab could have been resumed if LVEF returned to the normal limit and the absolute decrease from baseline was <15% within 4 to 8 weeks.

Assessments

Study visits took place every 3 weeks until week 16, then at weeks 25, 34, 40, and 49, and then every 12 weeks thereafter until the patient met discontinuation criteria or the study ended.

Adverse events were graded according to the National Cancer Institute Common Terminology Criteria for Adverse Events v4.03.

Online Resource 2

Statistical analysis

All variables are presented as descriptive statistics (number of patients, mean, median, standard deviation, minimum, and maximum) for continuous end points and as frequency and percentage for categorical end points.

Analysis sets

The safety analysis set (SAF) included all enrolled patients who received at least one or a partial dose of study treatment. The full analysis set (FAS) was defined as all patients in the SAF who had centrally assessed AR+ breast cancer (defined as ≥10% of tumor cells with nuclear expression). The efficacy evaluable set (EES) included all patients in the FAS who had at least one available post-baseline tumor assessment. The primary analysis was performed in the EES, while all efficacy analyses were performed in both the EES and FAS.

Online Resource 3

Fig. S1. Patient disposition

[a]Completed means one of the following: (i) patient progressed while on treatment and has completed the safety follow-up visit, (ii) patient is in disease progression follow-up and started a new antineoplastic therapy, and (iii) patient is in disease progression follow-up and reaches disease progression

AR androgen receptor, *EES* efficacy evaluable set, *FAS* full analysis set, *IV* intravenous, *Q21D* every 21 days, *QD* once a day, *SAF* safety analysis set, *SC* subcutaneous

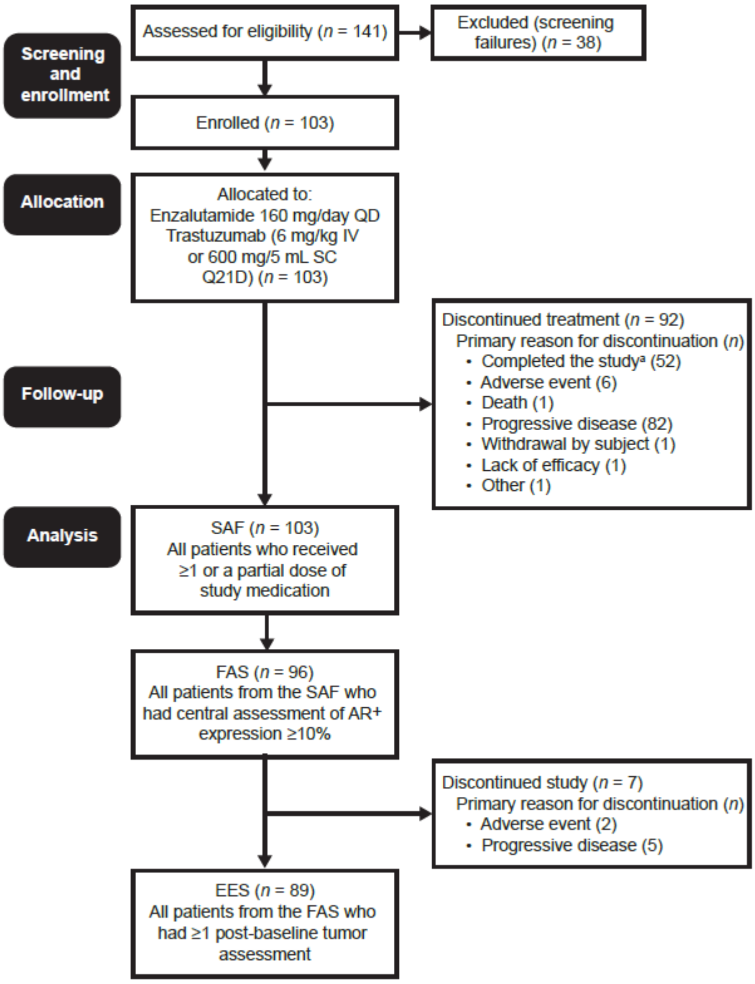

Online Resource 4

Table S1. Concordance of central and local AR test results

	Full analysis set combined with low-AR-expression set[a] (n = 98)			
	Central testing results			
	Positive	Negative	Unknown	Total
Local testing results, *n* (%)				
Positive	73 (74.5)	0	0	73 (74.5)
Negative	1 (1)	0	0	1 (1)
Unknown	24 (24.5)	0	0	24 (24.5)
Total	98 (100)	0	0	98 (100)
Concordance rate,[b] %	98.6			

AR androgen receptor

[a]Low-AR-expression set comprises enrolled patients who received at least one dose of study drug and had central assessment of AR+ expression >0% and <10%

[b]Concordance rate is 100 × the proportion of patients whose local and central results are both positive or both negative out of all patients with known (i.e., positive or negative) local and central results

Online Resource 5

Table S2. Study drug exposure

Study drug exposure	Safety set (n = 103)
Enzalutamide	
Duration of exposure (months), n (%)	
<3	56 (54.4)
≥3 to <6	24 (23.3)
≥6	23 (22.3)
Duration of exposure, days	
Mean (SD)	124.4 (114.1)
Median	70.0
Minimum, maximum	1, 660
Dose reduction, n (%)	
None	94 (91.3)
1	7 (6.8)
2	2 (1.9)
Dose interruption, n (%)	
None	87 (84.5)
1	12 (11.7)
2	4 (3.9)
Dose discontinuation, n (%)	
No	9 (8.7)
Yes	94 (91.3)

Trastuzumab	
Number of complete infusions administered per patient	
Mean (SD)	6.0 (5.2)
Median	4
Minimum, maximum	1, 30
Number of incomplete infusions administered	1
Reason for dose interruption/discontinuation, *n* (%)	
Infusion reaction	1 (1.0)
Other adverse event	0 (0.0)
Infusion equipment malfunction	0 (0.0)
Other	1 (1.0)

SD standard deviation

Online Resource 6

Table S3. Clinical benefit rate at 24 weeks by AR % and HR status subgroups

	Clinical benefit rate at 24 weeks, *n* (%[a])
Overlapping AR % nuclear staining groups (*n*)	**FAS and low-AR-expression set[b] (*n* = 98)**
>0 (98)	21 (21.4)
≥30 (93)	20 (21.5)
≥50 (88)	19 (21.6)
≥80 (77)	17 (22.1)
Mutually exclusive AR % nuclear staining groups (*n*)	
>0–<30 (5)	1 (20.0)
≥30–<50 (5)	1 (20.0)
≥50–<80 (11)	2 (18.2)
≥80 (77)	17 (22.1)
HR status[c] (*n*)	**EES (*n* = 89)**
HR+ (46)	12 (26.1)
HR– (31)	8 (25.8)
HR unknown (12)	1 (8.3)

AR androgen receptor, *EES* efficacy evaluable set, *ER* estrogen receptor, *FAS* full analysis set, *HR* hormone receptor, *PgR* progesterone receptor

[a]Percentages were calculated out of the total number of patients in each subgroup

[b]Low-AR-expression set consists of enrolled patients who received at least one dose of study drug and had central assessment of AR+ expression >0% and <10%

[c]Positive HR status = ER+ and PgR+ (n=23), ER+ and PgR– (n=18), or ER– and PgR+ (n=5)

Online Resource 7

Table S4. Clinical benefit rate at 24 weeks by previous lines of therapy

	Clinical benefit rate at 24 weeks, *n* (%[a])
Number of lines of prior of antineoplastic therapy (*n*)	**EES (*n* = 89)**
1–2 (28)	6 (21.4)
3–4 (21)	5 (23.8)
≥5 (40)	10 (25.0)
Number of lines of prior anti-HER2 therapy (*n*)	**EES (*n* = 88)**
1–2 (33)	7 (21.2)
3–4 (24)	8 (33.3)
≥5 (31)	6 (19.4)

EES efficacy evaluable set, *HER2* human epidermal growth factor receptor 2

[a]Percentages were calculated out of the total number of patients in each subgroup
